# Inhibitory Projections in the Mouse Auditory Tectothalamic System

**DOI:** 10.3390/brainsci8060103

**Published:** 2018-06-09

**Authors:** Blaise A. Clarke, Charles C. Lee

**Affiliations:** Department of Comparative Biomedical Sciences, LSU School of Veterinary Medicine, Baton Rouge, LA 70803, USA; bclark6@lsu.edu

**Keywords:** inferior colliculus, medial geniculate body, auditory, inhibition, GABA

## Abstract

The medial geniculate body (MGB) is the target of excitatory and inhibitory inputs from several neural sources. Among these, the inferior colliculus (IC) is an important nucleus in the midbrain that acts as a nexus for auditory projections, ascending and descending, throughout the rest of the central auditory system and provides both excitatory and inhibitory inputs to the MGB. In our study, we assessed the relative contribution from presumed excitatory and inhibitory IC neurons to the MGB in mice. Using retrograde tract tracing with cholera toxin beta subunit (CTβ)-Alexa Fluor 594 injected into the MGB of transgenic, vesicular GABA transporter (VGAT)-Venus mice, we quantitatively analyzed the projections from both the ipsilateral and contralateral IC to the MGB. Our results demonstrate inhibitory projections from both ICs to the MGB that likely play a significant role in shaping auditory processing. These results complement prior studies in other species, which suggest that the inhibitory tectothalamic pathway is important in the regulation of neuronal activity in the auditory forebrain.

## 1. Introduction

The medial geniculate body (MGB) is a critical part of the central auditory system, acting as a neural hub for ascending and descending axonal projections [1,2,3,4,5]. As the thalamic region responsible for audition, it is the gateway for acoustic input en route to the auditory cortex [6,7,8,9,10,11]. The MGB can be subdivided into three main regions (dorsal, ventral, and medial), composed of different neuronal cell types, including excitatory and inhibitory neurons in most species, except rodents that lack local interneurons [2,12,13]. The MGB has been studied extensively in terms of its anatomy, tonotopic organization, neuronal makeup, and connections to other auditory regions, especially the midbrain and cortex [2,14,15,16,17,18,19,20,21,22,23,24,25].

The major source of ascending input to the MGB arises from the inferior colliculus (IC), which integrates input from lower brainstem nuclei [5]. This tectothalamic projection is unique among ascending sensory systems, since it is composed of an inhibitory pathway from IC GABAergic cells, which represents ~20–40% of the neurons involved in the tectothalamic pathway [1,26,27,28]. The auditory tectothalamic pathway thus involves a convergence of excitatory and inhibitory neurons to the MGB, further underscoring its complexity. The inhibitory GABAergic tectothalamic pathway provides an additional avenue for inhibition of the MGB, besides local interneurons and projections from the thalamic reticular nucleus (TRN) [13,29,30]. With several sources of possible inhibition in the MGB, the consideration of the relative influence of how these multiple inhibitory projections interact with convergent ascending and descending excitatory inputs is critical to understanding the relative function of each input.

The goal of this project was to examine the contribution of the excitatory and inhibitory tectothalamic projections to the MGB in the mouse. Here, we used a vesicular GABA transporter (VGAT)-Venus, transgenic mouse model which enabled the rapid identification of inhibitory neurons in these structures [31,32]. We employed retrograde tract-tracing to identify the excitatory and inhibitory connections from the IC that terminate in the MGB. An additional objective for this project was to assess the proportional input from these sources to the MGB, quantitatively assessing the intensity of each based on retrograde-labeled neuronal count and to also confirm the type of projection involved, that is, excitatory or inhibitory.

## 2. Materials and Methods

### 2.1. Surgery

The following procedures were approved by the Institutional Animal Care and Use Committee (IACUC) of the Louisiana State University School of Veterinary Medicine. Transgenic mice (3 males and 3 females, ~5–6 months of age) (colony breeder mice kindly provided by Dr. Janice Naegele at Wesleyan University) expressing the Venus fluorescent protein (developed by Dr. Atsushi Miyawaki at RIKEN, Wako, Japan) in inhibitory, vesicular GABA transporter (VGAT)–positive neurons were used. These transgenic mice were raised on a C57BL/6J background. 

**Figure 1:**
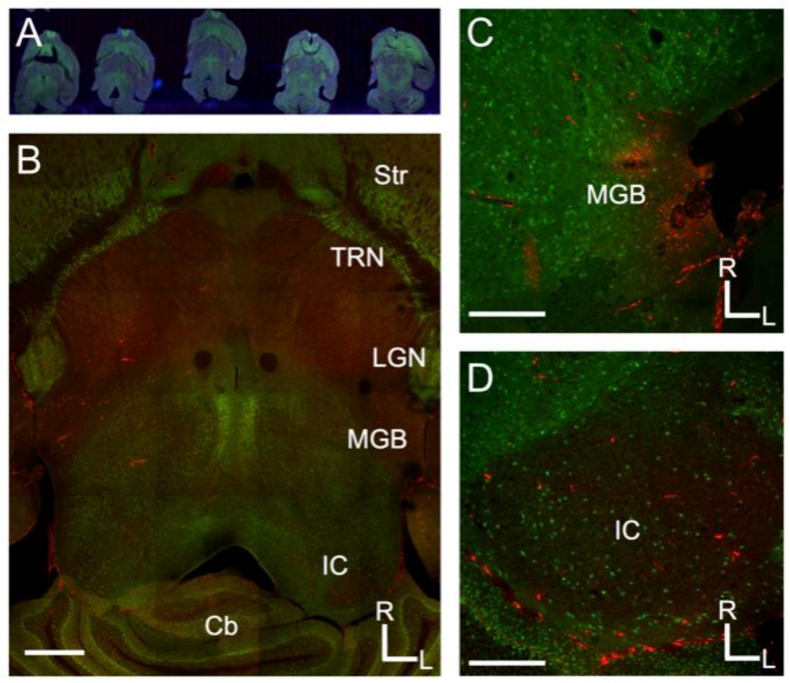
Projections from the inferior colliculus to the medial geniculate body in the mouse. (**A**) Overview of Nanozoomer scanned images from serial sections of the mouse brain sectioned to preserve the tectothalamic pathway (see Methods and Lee and Sherman, 2009); (**B**) Confocal image of a tectothalamic section showing the inferior colliculus (IC) and medial geniculate body (MGB). Other structures visible include the striatum (Str), thalamic reticular nucleus (TRN), lateral geniculate nucleus (LGN) and the cerebellum (Cb); (**C**) Example of injection site for CTβ-Alexa 594 in the lateral part of the MGB; (**D**) Labeling in the ipsilateral IC following the injection depicted in C. In each image, green label is the Venus-fluorescent protein and red-label is the CTβ-Alexa 594. Scale bars are 1 mm (**B**), 500 μm (**C**), 500 μm (**D**).

For surgery, the mice were anesthetized with a ketamine/xylazine mixture (0.1 mL/20 g) [31,32]. The head was shaved and cleaned with betadine prior to injection, then placed in a stereotaxic head-holder frame (Stoelting, Wood Dale, IL, USA). Body temperature was maintained at 37 °C with a heating pad. Sterile instruments and aseptic techniques were used throughout the surgery. An incision in the scalp and a craniotomy were made above the stereotactic target coordinates for the medial geniculate body (MGB) (relative to bregma: AP: −3.16 mm, ML +1.80 mm) [33]. Once the craniotomy was made, a Hamilton microsyringe with a sterile needle was lowered to target the ventral medial geniculate body (MGBv) (depths of 3.0 to 3.5 mm) and used to pressure inject Cholera Toxin Beta Subunit (CTβ) (Recombinant), Alexa Fluor^®^ 594 Conjugate (Thermofisher Scientific, Waltham, MA, USA), a fluorescent tracer dye, according to the manufacturer’s recommendations for retrograde tracing volume (~0.2–0.5 μL in the 6 animals used), typically spreading from ~25–50% of the volume of the MGB (Figure 1C). After the tracer dye was deposited and equilibrated, the animal’s scalp was sutured, and the animal was removed from the stereotaxic framework. Each animal was observed and monitored post-surgery until fully recovered.

### 2.2. Histology and Imaging

Each animal was then recovered for three to five days to allow for the tracer to transport optimally and were monitored for health during this period. After this transport period, the animal was deeply anesthetized with isoflurane and sacrificed via transcardiac perfusion, using 1ml of 10 mM phosphate-buffered saline (PBS) solution, followed by 6 mL of 4% paraformaldehyde (PFA) in 10 mM PBS fixative. The brain was then removed and postfixed in 4% PFA/in 10 mM PBS at 4 °C for 24 h, after which it was stored in a 4% PFA with 30% sucrose solution in 10 mM PBS for 24 h for cryoprotection at 4 °C. Subsequently, the brain was prepared for cryosectioning; the brain was blocked with a 35-degree cut on the dorsal surface and sliced to preserve the IC, MGB and AC, that is, the tectothalamic sectioning plane that we have previously described [29]. Using a cryostat, each brain was frozen sectioned at 50 μm, and sections collected in 48-well plates containing 10 mM PBS.

**Figure 2:**
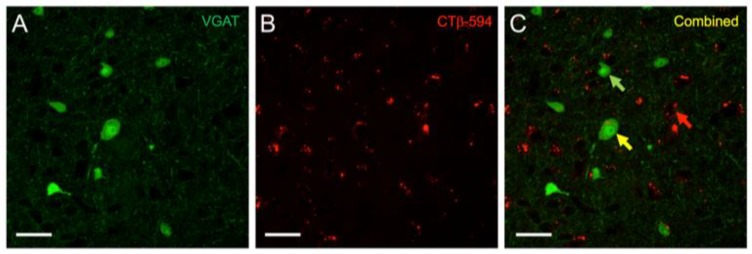
High-power image of vesicular GABA transporter (VGAT)-Venus and CTβ-Alexa 594 labeling in the IC. (**A**) Venus expression (green) was found in both large and small, disc-shaped and stellate neurons in the IC, which we have previously demonstrated to be primarily GABAergic [31]; (**B**) CTβ-Alexa 594 (red) labeling appeared as small vesicular shaped puncta within cell bodies; (**C**) The distinct appearance of the two labels enabled ready identification of retrograde-only (red arrow), Venus-only (green arrow), and double-labeled cells (yellow arrow).

A 1:2 series of sections was mounted and coverslipped on slides using Vectashield anti-fade mounting medium with 4′,6-diamidino-2-phenylindole (DAPI) (Vector Labs, Burlingame, CA, USA). Once the slides had dried, they were scanned using a Nanozoomer digital slide scanner (Figure 1A) (Hamamatsu, Naka-ku, Hamamatsu) and a FluoView microscope for confocal microscopy (Figure 1B) (Olympus, Center Valley, PA, USA), in preparation for the counting of retrogradely-labeled and Venus-expressing cells (Figure 1D and Figure 2). Neurons that exhibited labeled tracer were counted for quantitative analysis. Double-labeled (Venus+tracer) and single-labeled (tracer alone) cells were counted in both ipsilateral and contralateral IC. The cells were counted manually from processed images produced by the FluoView confocal microscope and a Nanozoomer digital slide scanner using Image J (NIH, Bethesda, MD, USA). An automatic counter analysis was also done via ImageJ for further confirmation of the results. The results of this analysis were used for an overall summary of the tracer’s distribution. Figures showing the distribution of the tracer throughout the aforementioned auditory regions were taken using Nanozoomer Digital Pathology (NDP).view2 (Hamamatsu) (Figure 1A).

## 3. Results

Retrograde tract tracing of convergent inputs to the MGB with CTβ-AlexaFluor 594 in VGAT-Venus mice showed a significant number of single- and double-labeled cells (Figure 1 and Figure 2). The VGAT-Venus transgenic mice are beneficial for readily identifying and tracing GABAergic circuitry, since they endogenously express an enhanced form of yellow fluorescent protein (YFP), the Venus protein, in inhibitory neurons [32]. We have previously demonstrated that the IC cells expressing the Venus protein from the VGAT promoter are almost entirely GABAergic (>95%) [31].

The CTβ injection sites displayed limited diffusion (<500 μm) of the tracer dye, similar to that seen in our prior studies [6,34,35], and generally labeled much of the lateral sector of the MGB (Figure 1C). Subsequent, retrograde labeling was found in both ICs, as well as the auditory cortex (AC), and the thalamic reticular nucleus TRN. 

Following these retrograde tracer injections in the MGB, we observed in the IC both single-labeled cells, identified as Venus-negative, retrograde-positive cells (Figure 2C: red arrow, red cell), and double-labeled cells, identified as VGAT-positive, retrograde-positive cells (Figure 2C: yellow arrow, red + green cell). In cells expressing the Venus fluorescent protein, the fluorophore was observed filling the entire cell body and the surrounding neurites (Figure 2A). Morphologically, we observed retrogradely-labeled, Venus-expressing cells that had both large and small somata, however the dendritic morphology was not readily apparent (Figure 2C). In contrast, the CTβ-retrograde fluorescent label appeared as round, vesicular-shaped puncta within the cell body. For single-labeled cells, the retrograde vesicular puncta were often localized in void-regions that were surrounded by Venus-positive puncta and neurites (Figure 2C: red arrow). We classified these single-labeled cells as non-GABAergic and were presumed glutamatergic, excitatory.

The distributions of single- and double-labeled retrograde cells were counted, from the confocal and Nanozoomer digital microscopy images (see Methods). The percentages of single- and double-labeled retrograde cells in both ICs were generally similar, but with approximately an order of magnitude more retrogradely-labeled cells counted in the ipsilateral IC (Table 1). Topographically, the distribution of the two types of retrogradely labeled cells were mostly overlapping, as many single-labeled cells were clustered adjacent to the double-labeled cells (Figure 1D and Figure 2C).

The ipsilateral inferior colliculus (IC) contained single- and double-labeled retrograde cells projecting to the medial geniculate body (MGB); 30 ± 2% of the ipsilateral IC’s retrograde cells were double-labeled, presumed GABAergic inhibitory, while the rest were VGAT-negative, presumed glutamatergic excitatory, as suggested based on previous studies [36,37,38,39]. The ipsilateral IC displayed a similar proportion of GABAergic (30 ± 2%) neurons labeled by the tracer dye, in comparison to the contralateral IC (36 ± 5%) (Table 1). These anatomical results further confirm past studies in other species that have found ascending GABAergic tectothalamic projections [1,26,27,28].

## 4. Discussion

In this study, we used retrograde tract tracing with the CTβ-AlexaFluor 594 tracer dye to quantitatively characterize the neuronal pathways arising from the IC that project to the MGB. Our data, using the VGAT-Venus mouse, reveal excitatory and inhibitory projections from the IC to the MGB. These data strengthen past findings of homologous regions in various species, such as the cat, rat, and guinea pig [1,26,27,28]. Our use of the VGAT-Venus transgenic mouse strain is particularly advantageous, since the native expression of the Venus-fluorescent protein in GABAergic neurons enabled the simple and relatively unambiguous identification of inhibitory neuronal cells [31,32], whereas prior studies utilized immunohistochemical detection of these cells [1,26,27,28]. Our assignment of these Venus-expressing cells as inhibitory is based on our previous data showing >95% overlap of GABA expression in the VGAT-Venus neurons in the IC [31], although this indicates that it is likely that some of our single-labeled cells could potentially be inhibitory. Although the Venus protein is also expressed in glycinergic cells in this transgenic line, it is unlikely that any of the tectothalamic projection is glycinergic, based on prior studies that have demonstrated that GABA is the neurotransmitter utilized in the auditory tectothalamic pathway [1,26,27,28,29,30,40].

One drawback with the VGAT-Venus mice in neuroanatomical studies of the auditory pathway are that they are raised on the C57BL/6J background, which exhibits a well-characterized, hearing loss at high frequencies beginning at 6 months of age [41]. In our studies, we attempted to mitigate this by using mice younger than this age.

Our results correlate with previous studies of the tectothalamic projection in cats [26], rats [1], and the guinea pig [27,28]. In general, these studies have noted a species-specific range in the proportion of inhibitory tectothalamic inputs, with cats and guinea pigs exhibiting roughly similar percentages ~20%, while rats are much higher, ~40%. By comparison, although our finding of ~30% of ipsilateral inhibitory tectothalamic neurons is somewhat midway between these two previously reported values, it may actually be considered more akin to that of the rat, since our values likely represent a slight underestimation of the total inhibitory tectothalamic projection, as noted above. As such, mice and rats may exhibit potentially greater inhibitory drive to the MGB from the IC than cats and guinea pigs [1,26,27,28].

**Figure 3:**
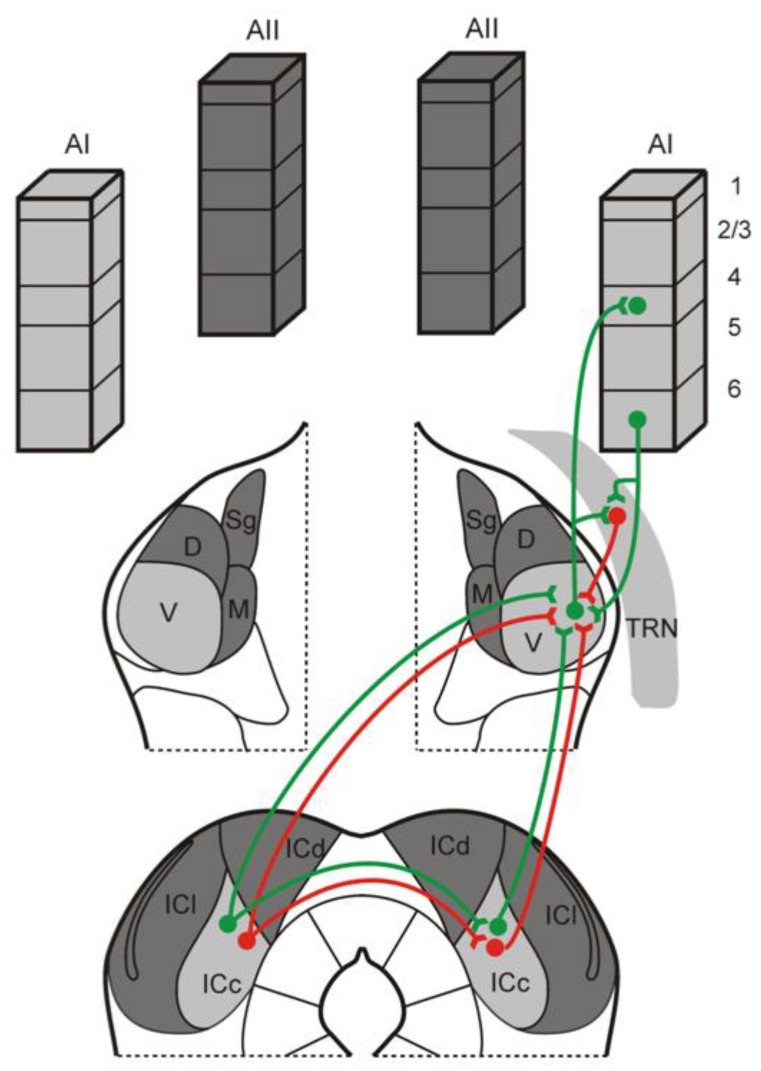
Schematic diagram illustrating the organization of ipsilateral and contralateral tectothalamic inputs to the IC. As demonstrated in this study, the mouse MGB receives both ascending excitatory (green) and inhibitory (red) inputs from the ipsilateral and contralateral IC. In addition, the contralateral IC could potentially influence the ipsilateral MGB via indirect projections through the IC commissure that target ascending ipsilateral tectothalamic projections [31]. The MGB itself integrates these inputs with inhibitory inputs from the thalamic reticular nucleus (TRN) and excitatory inputs from layer 6 of the auditory cortex (AC) [47,48,49,50].

The inhibitory tectothalamic projection is one of several potential sources of inhibition to MGB relay neurons, which also receive local inhibition from interneurons (in non-rodents) and feedback inhibition from the thalamic reticular nucleus (TRN) that is driven by the thalamus and corticothalamic neurons in layer 6 (Figure 3) [13,42,43,44,45]. The greater inhibitory tectothalamic contribution in rats, like mice, has been posited to compensate for their relative lack of interneurons [13,46], but which does not account for the relatively weaker inhibitory tectothalamic inputs in guniea pigs, which also lack local MGB interneurons [27,28]. As such, we speculate that the greater inhibitory tectothalamic projection in mice and rats may have evolved to suit potentially similar ethological requirements and evolutionary histories.

In addition, our study shows that the contralateral IC provides a less intense, but apparent inhibitory projection to the contralateral MGB, which may enable further ascending inhibitory control of auditory processing. The overall proportion was similar to that from the ipsilateral IC, but an order of magnitude fewer in number. Such a contralateral IC projection has been noted in other studies, and our results are similar in also exhibiting a crossed ascending inhibitory tectothalamic projection [5,26,27,28]. As such, there exist two potentially unique routes for contralateral control of the MGB by the IC: a direct contralateral tectothalamic pathway and an indirect tectotectothalamic pathway, composed of commissural IC inputs to ascending ipsilateral tectothalamic projections (Figure 3) [31]. Thus, contralateral interactions in the tectothalamic system represent a complex and largely overlooked mechanism enabling the integration of bilateral acoustic information [31].

## 5. Conclusions

In conclusion, these results demonstrate ascending excitatory and inhibitory inputs to the medial geniculate body from both the ipsilateral and contralateral inferior colliculi in the mouse, confirming similar findings in other species [1,26,27,28]. Given the growing importance of the mouse as a model system for investigations of auditory processing, particularly with regard to modern optogenetic and DREADD methodologies, these data provide an anatomical framework for future studies of the physiological, computational, and behavioral roles of the auditory tectothalamic pathways in the mouse. 

## Figures and Tables

**Table 1:** Quantitative analysis of retrogradely labeled GABAergic and non-GABAergic projections to the MGB in the VGAT-Venus mouse from the ipsilateral and contralateral inferior colliculus (IC). Total number of counted cells from 6 animals indicated in column headings. VGAT+ and VGAT− retrograde cells values (mean ± s.d.) are shown as percentages of total excitatory and/or inhibitory neurons in that region.

	Ipsi IC (*n* = 3650)	Contra IC (*n* = 297)
Mean VGAT+ retrograde cells (%)	30 ± 2	36 ± 5
Mean VGAT− retrograde cells (%)	70 ± 2	64 ± 5
